# Cytosolic escape of mitochondrial DNA triggers cGAS-STING-NLRP3 axis-dependent nucleus pulposus cell pyroptosis

**DOI:** 10.1038/s12276-022-00729-9

**Published:** 2022-02-10

**Authors:** Weifeng Zhang, Gaocai Li, Rongjin Luo, Jie Lei, Yu Song, Bingjin Wang, Liang Ma, Zhiwei Liao, Wencan Ke, Hui Liu, Wenbin Hua, Kangcheng Zhao, Xiaobo Feng, Xinghuo Wu, Yukun Zhang, Kun Wang, Cao Yang

**Affiliations:** Department of Orthopaedics, Union Hospital, Tongji Medical College, Huazhong University of Science and Technology, Wuhan, 430022 China

**Keywords:** Cell death, Diseases

## Abstract

Low back pain (LBP) is a major musculoskeletal disorder and the socioeconomic problem with a high prevalence that mainly involves intervertebral disc (IVD) degeneration, characterized by progressive nucleus pulposus (NP) cell death and the development of an inflammatory microenvironment in NP tissue. Excessively accumulated cytosolic DNA acts as a damage-associated molecular pattern (DAMP) that is monitored by the cGAS-STING axis to trigger the immune response in many degenerative diseases. NLRP3 inflammasome-dependent pyroptosis is a type of inflammatory programmed death that promotes a chronic inflammatory response and tissue degeneration. However, the relationship between the cGAS-STING axis and NLRP3 inflammasome-induced pyroptosis in the pathogenesis of IVD degeneration remains unclear. Here, we used magnetic resonance imaging (MRI) and histopathology to demonstrate that cGAS, STING, and NLRP3 are associated with the degree of IVD degeneration. Oxidative stress induced cGAS-STING axis activation and NLRP3 inflammasome-mediated pyroptosis in a STING-dependent manner in human NP cells. Interestingly, the canonical morphological and functional characteristics of mitochondrial permeability transition pore (mPTP) opening with the cytosolic escape of mitochondrial DNA (mtDNA) were observed in human NP cells under oxidative stress. Furthermore, the administration of a specific pharmacological inhibitor of mPTP and self-mtDNA cytosolic leakage effectively reduced NLRP3 inflammasome-mediated pyroptotic NP cell death and microenvironmental inflammation in vitro and degenerative progression in a rat disc needle puncture model. Collectively, these data highlight the critical roles of the cGAS-STING-NLRP3 axis and pyroptosis in the progression of IVD degeneration and provide promising therapeutic approaches for discogenic LBP.

## Low back pain: Understanding disc degeneration yields possible therapeutic target

An investigation of the mechanisms responsible for intervertebral disc degeneration (IVD) reveals a potential treatment to limit the progression of low back pain. IVD involves widespread inflammation and cell death inside discs, but exactly how the condition progresses is unclear. Cao Yang, Kun Wang and co-workers at Huazhong University of Science and Technology, Wuhan, China, investigated IVD in patient samples and rat models. Oxidative stress triggered leakage of mitochondrial DNA into cellular fluid in cells in the center of discs. This activated an immune response pathway and prompted the release of inflammatory cytokines. The degree of activation of this pathway is directly linked to IVD severity, providing a potential method of monitoring the condition. Administering a drug to rats to block mitochondrial DNA leakage slowed IVD progression.

## Introduction

Low back pain (LBP) is a major musculoskeletal disorder and the leading cause of disability worldwide with a high prevalence^1–4^. Globally, LBP is the second most common reason for medical consultation in industrialized countries^5,6^. It is now well established that LBP is mainly involved in intervertebral disc (IVD) degeneration^3,7^. IVDs are complex avascular fibrocartilaginous tissues consisting of nucleus pulposus (NP), annulus fibrosus (AF), and cartilaginous endplates, which connect adjacent vertebral bodies to absorb spine axial compressive forces and to facilitate load transmission^8,9^. As the hydrogel-like core of the IVD, healthy NP tissue is primarily composed of NP cells and extracellular matrix (ECM), and its elastic property distributes hydraulic pressure in all directions within each IVD^10^. Resident NP cells are critical for NP tissue function and ECM metabolism to maintain the gelatinous properties of NP tissue^11^.

The process of IVD degeneration is complicated by the occurrence of three intertwined events that form a vicious cycle: (i) the progressive loss of NP cells, (ii) inflammation, catabolic cascades, and ECM dehydration, and (iii) declines in cellular functions and biomechanical properties^8,12–14^. The loss of resident NP cells is likely to be one of the initiating events in IVD degeneration that leads to the formation of an inflammatory microenvironment and an imbalance in ECM metabolism, ultimately contributing to the onset of a degenerative process with decreasing biomechanical properties and discogenic LBP^8,9,11,15^. Therefore, an improved understanding of the molecular mechanism underlying NP cell loss might provide new targets to improve the treatment of IVD degeneration.

Excessively accumulated cytosolic DNA acts as a kind of pathogen- or damage-associated molecular pattern (PAMP and DAMP) that is sensed by pattern recognition receptors (PRRs) to trigger the immune response^16,17^. The cGAS-STING axis is composed of cyclic GMP-AMP synthase (cGAS) and cyclic GMP-AMP receptor stimulator of interferon genes (STING) and responds to pathogens or damaged DNA as a crucial immune axis in microbial infection, chronic inflammation, cancer progression and organ degeneration^17–21^. Cellular stress can disrupt mitochondrial homeostasis, promoting mitochondrial permeability transition pore (mPTP) opening and mitochondrial DNA (mtDNA) efflux into the cytosol, which can trigger inflammatory responses through the cGAS-STING signaling axis and subsequently activate the IRF3 or NF-κB pathway^18,22–24^. The surveillance of mtDNA homeostasis by the cGAS-STING signaling axis is a key process in the response to cellular stress^16,20^.

The NLR pyrin domain containing 3 (NLRP3) inflammasome is a cytosolic protein complex that senses exogenous pathogenic patterns, endogenous damage signals and microenvironmental perturbations to maintain tissue homeostasis^25,26^. The NLRP3 inflammasome, which is composed of a sensor (NLRP3), the adaptor apoptosis-associated speck-like protein containing a CARD (ASC) and the effector caspase-1 (CASP-1), acts as a platform for CASP-1 activation, leading to the release of inflammatory cytokines and gasdermin D (GSDMD)-mediated cell pyroptosis^27–29^. Pyroptosis is a key type of inflammatory programmed death that is characterized by inflammasome activation, the formation of multiple transmembrane pores, cellular swelling with large bubbles and the leakage of cell contents to stimulate inflammation in response to pathogen- or damage-derived stress^27,30^. NLRP3 inflammasome-dependent pyroptosis leads to NP cell death and the formation of an inflammatory microenvironment, promoting a chronic inflammatory response and NP tissue degeneration^25^.

In this study, we revealed that cGAS, STING, and NLRP3 expression levels were increased in degenerative NP tissues. Importantly, we showed that mtDNA leaking into the cytosol via mPTP acted as a DAMP and that activation of the cGAS-STING-NLRP3 axis was responsible for NP cell pyroptosis induced by oxidative stress. Furthermore, experiments performed in vitro and in vivo showed that inhibiting mPTP opening and blocking the activation of STING could alleviate NP cell pyroptosis and degenerative progression, which indicates that mPTP opening, excessive accumulation of cytosolic mtDNA, and cGAS-STING-NLRP3 signaling axis-dependent pyroptosis are promising therapeutic drug targets for treating degenerative IVD progression.

## Materials and methods

### Ethics statement

The use of human medical records, imaging, and samples from human subjects was consistent with the Declaration of Helsinki. The Ethics Committee of Tongji Medical College, Huazhong University of Science and Technology approved all experimental protocols related to human samples and animal experiments (No. S341) (No. S2394).

### Study subjects

We included volunteers who underwent spine surgery because of idiopathic scoliosis or lumbar disc herniation without infection or tumor, immune or endocrine diseases in this study. In addition, we used the Pfirrmann MRI grading system to assess the degree of degeneration based on the patients’ symptoms, signs, and magnetic resonance images (MRI)^31,32^. A total of 16 patients’ NP tissue samples were collected, 9 males and 7 females aged 12-58 years. We defined 4 tissues from volunteers as Grade I because of idiopathic scoliosis, while the other 12 tissues were defined as Grade II-IV because the participants had lumbar disc herniation in different segments. Further information on the participants is provided in Supplementary Table 1. All of the samples were separated into two parts, frozen using liquid nitrogen for protein expression analysis for western blotting and fixed with 4% formaldehyde buffer to assess the histological degree of degeneration. In particular, the NP tissues of volunteers with idiopathic scoliosis were regarded as healthy for human NP cell investigation.

### Culture of human NP cells

The healthy samples described above were partly used to isolate human NP cells. Briefly, we used phosphate-buffered saline (PBS) to transport NP tissue, isolated cells, and plated the cells at 37 °C and 5% CO_2_ in 1:1 DMEM/F12 in combination with 15% fetal bovine serum (FBS, Gibco, USA) and 1% penicillin/streptomycin (Servicebio). We used fluorescent-activated cell soring for NP cell marker determination (CD24, 311117; KRT18, 628404; Biolegend). The second passage cells were used for further in vitro experiment^33,34^. NP cells were exposed to 100 μM or 200 μM tert-butyl-hydroperoxide (TBHP) in medium for 24 h in vitro, and vehicle treatment was used for the controls^14,35,36^. Treatment with 1 μM H-151 (MedChemExpress) or 10 μM cyclosporine (CsA) (MedChemExpress) with 100 μM TBHP for 24 h was used to test the effects of STING activation inhibition and blockade of mPTP opening in NP cells under oxidative stress.

### Western blotting

Frozen human tissues were homogenized, lysed, and extracted, and cells were lysed by RIPA (Beyotime) for 15 min and centrifuged to collect the proteins. Proteins (15–80 μg) were separated by 8–12% SDS-PAGE, transferred, blocked, washed, and incubated with specific primary antibodies and secondary antibodies. Information on the antibodies is presented in Supplementary Table 2. The reagents used for western blotting are presented in Supplementary Table 3. A ChemiDoc-It 610 Imaging System (UVP, USA) was used to detect and visualize the protein bands, and GAPDH was used for normalization.

### Histological staining and immunohistochemistry

We cut the tissue paraffin blocks into 4-μm slices and used hematoxylin and eosin (H&E) staining to evaluate the histological degree of degeneration of human samples. Furthermore, sections were labeled with the primary antibody at 4 °C overnight, washed, and probed with the secondary antibody at room temperature for 1 h for immunohistochemical analysis.

### Immunofluorescence with tyramide signal amplification

Formalin-fixed paraffin-embedded (FFPE) human NP sections as described above were stained with fluorescent multiplex immunohistochemistry for analysis of the expression levels of cGAS and STING^37,38^. FFPE NP slides were deparaffinized by xylene, rehydrated by an ethanol gradient, and antigen retrieved by autoclaved Trilogy buffer (CellMarque) for 15 min. The sections were permeabilized by 0.5% Triton X-100 and blocked with 5% BSA. The sections were probed at 4 °C overnight with primary antibody, washed, and incubated for 1 h at 37 °C with the secondary antibody. Then, the sections were labeled with the tyramide-conjugated fluorophore for 10 min. Before the next primary antibody incubation, the sections were heated in 10 mM citric acid for 10 min to wash away the previous antibody. Antibodies paired with Opal tyramide-conjugated fluorophore were used as follows: cGAS and Opal 570, STING and Opal 520. The nuclei were labeled by 0.1 g/ml DAPI. Slices were captured under the Vectra 3.0 multispectral imaging system (PerkinElmer).

### RNA interference

For STING knockdown, we used Lipofectamine 2000 (Invitrogen) to transfect NP cells for 72 h with 100 nM STING small-interfering RNA (si-STING, GenePharma) or the control and immediately exposed them to 100 μM TBHP. The siRNA sequences are presented in Supplementary Table 4.

### General PCR and RT-qPCR

Cellular RNA was collected by TRIzol reagent (Invitrogen), and RNA reverse transcription to cDNA was performed. General PCR was performed, followed by digital imaging of agar gelatin electrophoresis to visualize the DNA abundance. RT-qPCR was conducted to quantitatively analyze the DNA content. Expression levels were normalized against that of GAPDH. The set of deltaCq replicates for the control and tested samples normalized against the geometric means of the reference genes was used for statistical testing and estimation of the *P* values. The primers used for PCR or RT-qPCR are presented in Supplementary Table 4.

### Measurement of cytosolic mtDNA

After induction of TBHP for the indicated time and dose, cytosolic mtDNA was analyzed by general PCR or RT-qPCR^18,23^. Briefly, half of the cells were lysed by mild lysis buffer, and the other half was lysed by strong lysis buffer. Cells were cytoplasmically lysed with 0.1% NP-40 for 20 min on ice and centrifuged at 14,000 × *g* for 20 min at 4 °C. Cytosolic mtDNA from the supernatant cytosolic fraction and total mtDNA from the total lysis were purified by a TIANamp Genomic DNA Kit. General PCR followed by agar gelatin electrophoresis and RT-qPCR as described above were used for DNA analysis normalized by the mtDNA in the total lysate.

### Measurement of cytosolic or mitochondrial reactive oxygen species (ROS)

DCFH-DA (Beyotime) and MitoSOX (Yeasen Biotech) were used as markers to measure cytosolic and mitochondrial ROS. After the treatment, the samples were stained according to the protocols described. The fluorescence intensities of DCFH-DA were detected by flow cytometry, while those of MitoSOX were visualized by fluorescence microscopy.

### Assessment of mitochondrial membrane potential (MMP) and mPTP opening

MMP was measured by a JC-1 Assay Kit (Beyotime) according to the protocol described, detected by a flow cytometer, and analyzed by the ratio of JC-1 polymers to monomers. mPTP was detected by an mPTP Assay Kit (Beyotime). Briefly, the treated cells were washed with PBS and incubated with calcein AM plus Co^2+^ quencher at 37 °C for 30 min. Then, the dye was replaced by culture medium, and the slides were cultured at 37 °C for 30 min in the dark and observed by a fluorescence microscope.

### Immunofluorescence

Cultured NP cells were fixed, permeabilized, blocked, incubated with primary antibodies at 4 °C overnight, washed thoroughly, and labeled with secondary antibodies at 37 °C for 1 h protected from light. Information on the antibodies is presented in Supplementary Table 2. Nuclei and mitochondria were stained with 0.1 g/ml DAPI (Beyotime) and 50 mM MitoTracker Red CMXRos (Invitrogen).

### Assessment of human NP cell viability

Adherent cells were washed thoroughly after treatment and probed with 5 μg/ml Hoechst 33342 (Invitrogen) and 5 μg/ml propidium iodide (PI, Invitrogen) for 20 min at 37 °C protected from light. After three washes, the adherent cell slices were captured by fluorescence microscopy.

### Human IL-1β ELISA (human interleukin-1β enzyme-linked immunosorbent assay)

After the treatment, cell culture supernatants were collected, secretory IL-1β was detected by an ELISA Kit (Elabscience), and the absorbance at 450 nm was measured by SoftMax Pro version 5 Software (Molecular Devices).

### CASP-1 enzymatic activity assay

A CASP-1 Assay Kit (Beyotime) was used to detect the enzymatic activity of CASP-1. After treatment, cellular proteins were extracted and coincubated with 2 mM acetyl-Tyr-Val-Ala-Asp p-nitroanilide (Ac-YVAD-pNA) at 37 °C for 24 h. The amount of p-nitroanilide (pNA) cleaved by CASP-1 was detected by a spectrophotometer at 450 nm, and the enzyme activity was calculated according to Chemicon’s unit normalized to the cellular total protein amount.

### DNA immunoprecipitation

After treatment, the cells were lysed with moderate IP buffer (NP-40, Beyotime) with 1% protease inhibitors (Servicebio) and 1.5% phosphatase inhibitor (MedChemExpress) at 4 °C for 20 min and centrifuged at 14,000 × *g* for 15 min at 4 °C to separate the soluble fraction. The resultant supernatants were incubated with anti-dsDNA or IgG isotype control monoclonal antibody at 4 °C for 8 h. Then, 1 mg protein was incubated with 5 μg antibodies. Protein A/G magnetic beads (MedChemExpress) were coincubated with the antigen-antibody mixture for 2 h at 4 °C. The beads were washed and divided into two parts. Half of the beads were eluted with SDS loading buffer (Boster) and then boiled at 95 °C for 10 min for western blotting analysis, while the other half were eluted with chromatin immunoprecipitation (ChIP) elution buffer (5 mM Tris-HCl, 10 mM EDTA and 1% SDS). After proteinase K treatment, DNA was collected using a TIANamp Genomic DNA Kit (Tiangen), followed by general PCR analysis as described above^18,39^.

### Proximity ligation assay (PLA)

After treatment, cells were fixed at room temperature for 15 min and washed twice with PBS. Then, the Duolink® In situ PLA ® kit (Sigma-Aldrich) for mouse/rabbit (with red detection) was used to show the physical interaction of dsDNA (double-stranded DNA) and cGAS according to the protocol.

### Transmission electron microscopy (TEM)

After treatment, cultured cells were successively fixed with 2.5% glutaraldehyde (Sigma-Aldrich) for 1 h and 2% osmium tetraoxide for 2 h. After washes with double distilled water, the cell samples were stained with 0.5% uranyl acetate for 12 h, dehydrated, polymerized, and cut into 70-90 nm ultrathin sections. Images were observed and captured by a Tecnai G2 TWIN TEM (FEI, USA).

### Needle puncture animal model and drug treatment

We used Sprague-Dawley rats (three months old, 200 ± 20 g) from the Laboratory Animal Center of Huazhong University of Science and Technology and housed the rats with a 12 h:12 h light:dark cycle at 21 °C. Pentobarbital (2%, 40 mg/kg) was used to anesthetize the rats (w/v). As previously described, the needle puncture-induced IVD degeneration model was established by using 29-gauge needle-to-percutaneous disc puncture on the Co8/9 and Co9/10 discs of each rats^32,40,41^. The needle vertical to the tail was inserted into the disc, punctured into at least 5 mm depth of disc, rotated through 360°, and kept in the disc for 60 s to ensure the degenerative effects. To avoid individual differences, we defined the punctured Co8/9 disc of each rats as the degenerated + PBS group (*n* = 7), the punctured Co9/10 disc of the same rat as the degenerated + CsA group (*n* = 7) or degenerated + C-176 group (*n* = 7), and the nonpunctured Co7/8 disc of the same rat as the negative control group (*n* = 7). For evaluation of the effects of cytosolic mtDNA and the cGAS-STING axis on IVD degeneration and the therapeutic potential of CsA and C-176 administration in vivo, punctured Co9/10 discs were immediately intradiscally injected with CsA (100 μM) or C-176 (10 μM) every week for one month, while other punctured Co8/9 discs were injected with the same amount of PBS every week for one month. To eliminate the influences of the injected volume, we injected 2 μl of the solution of interest into the center of the NP^42^.

### X-ray, micro-CT, and MRI examination

After one month of surgery, X-ray, micro-CT, and MRI analyses were performed on all rats before sacrifice. After anesthetization, the rats were kept in a supine position with their tail placed in a straight line. The X-ray, micro-CT, and MRI parameters are presented in Supplementary Table 5. We used NRecon to create three-dimensional reconstructions of micro-CT scans. The disc height index (DHI) and MRI degree of degeneration according to the Pfirrmann grade were determined as described previously.

### Histological analysis and immunofluorescence of rat model

After radiological examination, the rat samples were collected, fixed, decalcified, dehydrated, embedded in paraffin, and cut into 4 μm sections. H&E staining, safranin O-fast green staining, and Masson staining were conducted to assess the histological degree of degeneration of rat discs^43^. Immunofluorescence staining was carried out to analyze the expression levels of cGAS, STING, and NLRP3.

### Statistical analysis

Data are shown as the mean ± SEM. The number of experimental replicates and the level of significance are presented in the figure legends. GraphPad Prism software 8.0 was used to generate charts and perform statistical tests. We used Student’s *t* test and two-way ANOVA to determine significant differences between two groups and multiple groups. The *P* value was indicated by stars: n.s. no significance, *P < 0.05, **P < 0.01, ***P < 0.001.

More supplementary materials and methods are available in Supplementary Table 6.

## Results

### IVD degeneration was accompanied by the activation of the cGAS-STING axis and NLRP3 inflammasome

**Fig. 1:**
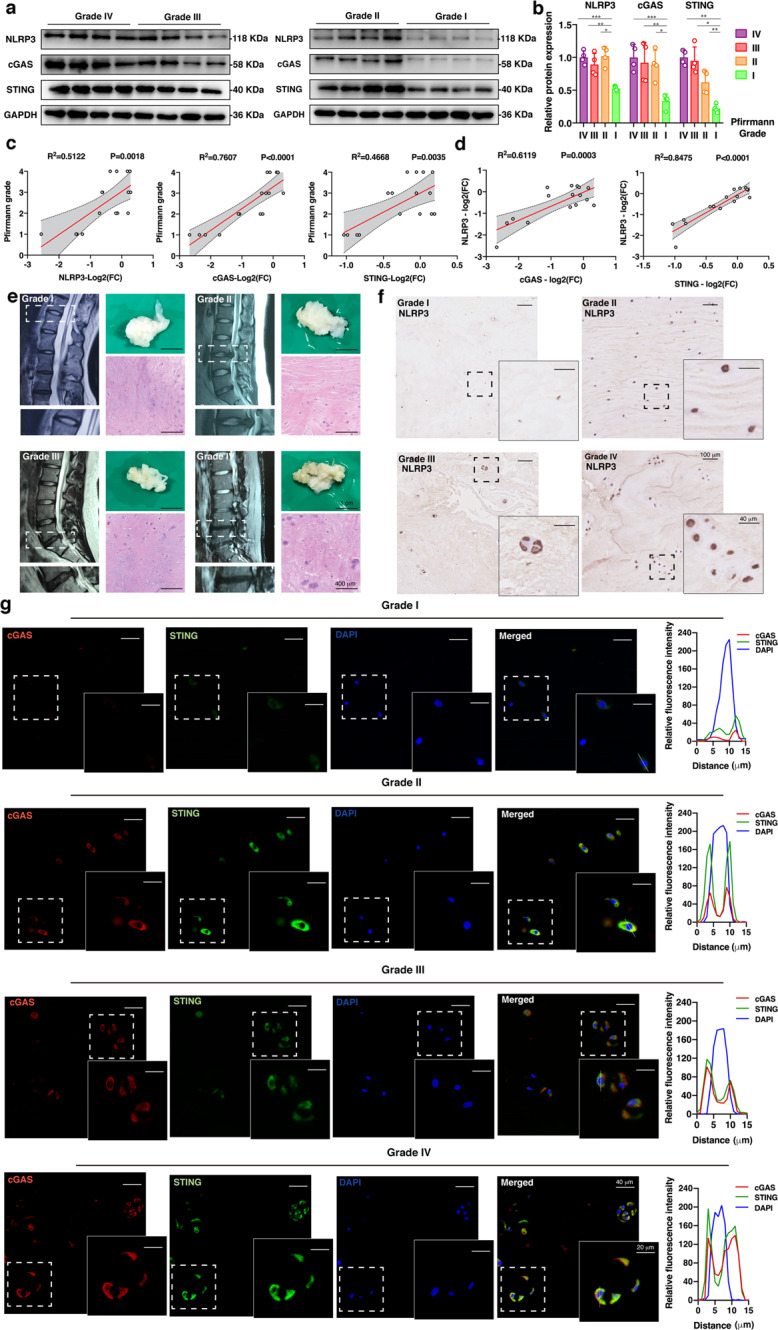
IVD degeneration was accompanied by the activation of the cGAS-STING axis and NLRP3 inflammasome. **a** Representative western blotting images of NLRP3, cGAS, and STING in the NP tissues of patients with different degrees of degeneration according to the Pfirrmann MRI grading system (*n* = 4). **b** Quantification of the protein expression levels of NLRP3, cGAS, and STING in the NP tissues of patients with different degrees of degeneration (*n* = 4). **c** Linear regression analysis between the NLRP3, cGAS, or STING protein levels (Log2 (FC): The log2-fold-change of the NLRP3, cGAS, or STING protein expression level compared to the average of Grade IV tissues) and the Pfirrmann MRI grades (*n* = 16). **d** Linear regression analysis between the cGAS or STING and NLRP3 protein levels in the NP tissues of patients (*n* = 16). **e** Representative MRI images, pathological images, and H&E staining of NP tissues with different degrees of degeneration. **f** Immunohistochemistry of NLRP3 in NP tissues with different degrees of degeneration. **g** Tyramide signal amplification immunofluorescence of cGAS and STING in the NP tissues of patients with different degrees of degeneration. Data are shown as the mean ± SD of at least three independent experiments. Statistical analyses were conducted using two-way ANOVA and Student’s *t* test. The *P* value is indicated by stars: *P < 0.05, **P < 0.01, ***P < 0.001.

To examine the roles of the cGAS-STING axis and NLRP3 inflammasome in IVD degenerative progression, we collected tissue samples from patients who underwent open spine surgery or lumbar discectomy surgery because of idiopathic scoliosis or lumbar disc herniation. According to the Pfirrmann MRI grading system, healthy tissues or disc tissues with different degrees of degeneration exhibited varied signal intensities by T2-weighted MRI. This result indicates a decrease in the water content of NP tissues with the progression of IVD degeneration^31^. Specifically, NP tissues with higher degrees of degeneration had atrophic volumes and decreased elasticity compared to those of tissues with lower degrees of degeneration. In addition, H&E staining showed that degenerated NP tissues exhibited clustered cellularity and chondroid nests with loss of the well-organized structure of the nucleus matrix, especially in Grade III and Grade IV tissues^43,44^ (Fig. 1e). Western blot analysis indicated that the protein expression levels of cGAS, STING, and NLRP3 were higher in the degenerated NP tissues (Fig. 1a, b). Linear regression analysis of cGAS, STING, or NLRP3 protein expression levels and degenerative IVD grades revealed that higher cGAS, STING, and NLRP3 protein levels were associated with worsened IVD degeneration (Fig. 1c). We further analyzed the relationship between cGAS or STING and NLRP3 protein levels, and a significant positive correlation was observed (Fig. 1d). Immunohistochemistry (IHC) showed that the protein expression levels of NLRP3 increased with the progression of IVD degeneration (Fig. 1f). Tyramide signal amplification immunofluorescence (TSA-IF) further revealed that cGAS and STING were increased and colocalized in the cytoplasm of degenerative NP tissues (Fig. 1g). These results revealed that IVD degeneration was accompanied by the activation of the cGAS-STING signaling axis and NLRP3 inflammasome.

### Oxidative stress triggered activation of the cGAS-STING axis and NP cell pyroptosis

**Fig. 2:**
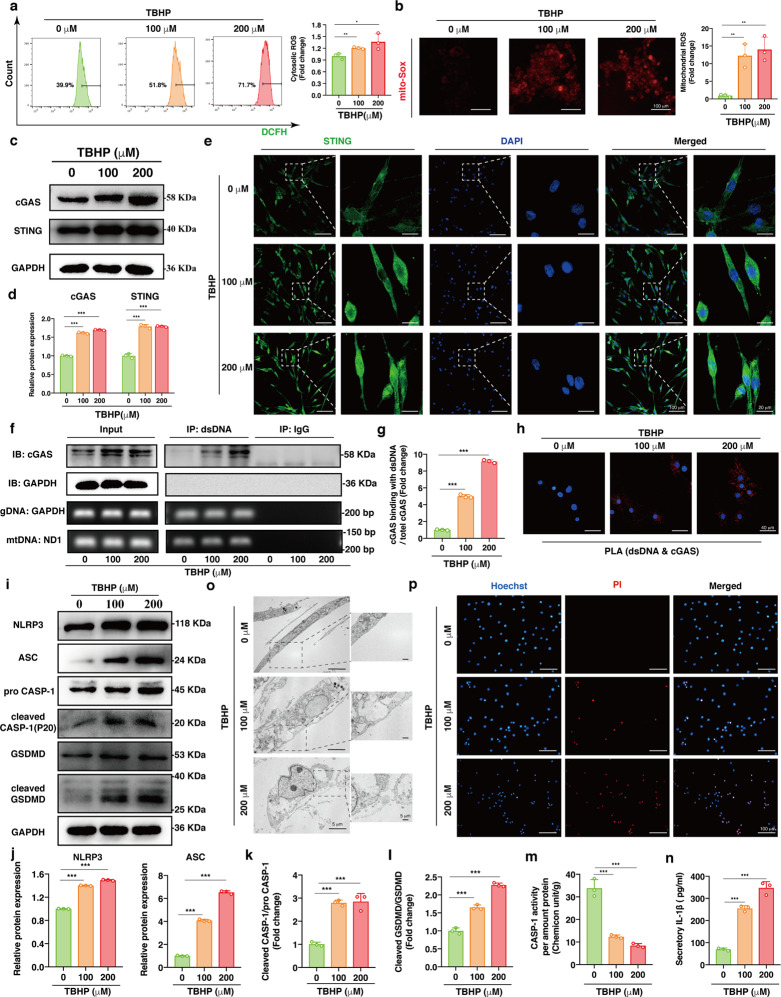
Oxidative stress triggered activation of the cGAS-STING axis and NP cell pyroptosis. Human NP cells were cultured with 0, 100 and 200 μM TBHP for 24 h. **a** Representative scatter plots of DCFH-DA staining and the quantitative analysis of green fluorescence for cytosolic ROS in the TBHP-treated NP cells compared to the controls. **b** Representative fluorescence images with MitoSOX and the quantitative analysis of fluorescence intensity for mtROS in NP cells exposed to TBHP compared to the controls. **c** Representative western blotting images of cGAS and STING in the TBHP-treated NP cells. **d** Quantification of the protein expression levels of cGAS and STING in the TBHP-treated NP cells compared to the controls. **e** Representative immunofluorescence images of STING with different degrees of polymerization in the TBHP-treated NP cells. **f** Representative DNA immunoprecipitation images, including western blotting images and DNA agar gelatin electrophoresis images, of the TBHP-treated NP cells (dsDNA: double-stranded DNA, gDNA: genomic DNA, mtDNA: mitochondrial DNA). **g** Quantification of the enrichment level of cGAS in DNA immunoprecipitates in the TBHP-treated NP cells (the ratio of cGAS pulled down by dsDNA antibody to total cGAS normalized to the average of the control). **h** In situ PLA was conducted to demonstrate the physical interaction between dsDNA and cGAS in the TBHP-treated NP cells. **i** Representative western blotting images of NLRP3, ASC, pro-CASP-1, cleaved CASP-1, GSDMD, and cleaved GSDMD in the TBHP-treated NP cells. **j** Quantification of the protein expression levels of NLRP3 and ASC in the TBHP-treated NP cells. **k** The ratio of cleaved CASP-1 to pro-CASP-1 in the TBHP-treated NP cells compared to the controls. **l** The ratio of cleaved GSDMD to GSDMD in the TBHP-treated NP cells compared with the controls. **m** The enzymatic activity of CASP-1 in the TBHP-treated NP cells (Chemicon unit/g: the amount of enzyme that could cleave 1 nmol Ac-YVAD-pNA to produce 1 nmol pNA at 37 °C for 1 h when the substrate was saturated) normalized to the total protein amount. **n** The quantitative level of IL-1β in cell culture supernatants after TBHP treatment. **o** TEM images of the TBHP-treated NP cells. **p** Representative fluorescence images of Hoechst/PI staining in the TBHP-treated NP cells. Data are shown as the mean ± SD of at least three independent experiments. Statistical analyses were conducted using two-way ANOVA and Student’s *t* test. The *P* value is indicated by stars: *P < 0.05, **P < 0.01, ***P < 0.001.

Previous studies have revealed that cellular oxidative stress plays a critical role in cell death and the microenvironmental imbalance in the progression of IVD degeneration^13,32^. To further investigate the cellular response under oxidative stress, we cultured human NP cells with different concentrations of TBHP for 24 h. Here, the cytosolic and mitochondrial ROS levels were probed by DCFH-DA and MitoSOX. We observed an increase in intracellular and mitochondrial ROS in the NP cells cocultured with TBHP for 24 h (Fig. 2a, b).

To determine whether NP cells activated the cGAS-STING axis under oxidative stress, we performed western blotting and showed a dose-dependent increase in cGAS and STING protein expression in the NP cells after TBHP treatment (Fig. 2c, d). Once bound to the second messenger cGAMP, the STING homodimer will close, release the C-terminal tail and expose the polymerization interface, which contributes to the formation of polymers via the cysteine-148 residue disulfide linkage^45^. Thus, immunofluorescence staining showed increased protein levels of STING with different degrees of polymerization and increased STING localization in the cytoplasm of NP cells after TBHP treatment (Fig. 2e), and quantitative analysis of the fluorescence intensity is shown in Supplementary Fig. 1a. Furthermore, to determine whether the interaction between dsDNA and cGAS increased in NP cells under oxidative stress, we immunoprecipitated DNA followed by western blotting to measure the coprecipitated proteins, and the results showed significant dose-dependent enrichment of cGAS in the TBHP-treated groups (Fig. 2f, g). In situ PLA was used to demonstrate the physical interaction between dsDNA and cGAS in NP cells, and PLA signals showed a significant dose-dependent increase after TBHP treatment (Fig. 2h). These data indicated that the cGAS-STING axis was activated in NP cells under oxidative stress.

Cellular stress-mediated NLRP3 inflammasome activation initiates pyroptotic cell death, triggering GSDMD-mediated transmembrane pore formation and the release of interleukin-1β (IL-1β) to form an inflammatory microenvironment^25,46^. To determine whether NLRP3 inflammasome activation and pyroptosis were responsible for NP cell death under oxidative stress, we used western blotting to examine the expression levels of key pyroptosis molecules and observed that NLRP3, ASC, pro-CASP-1, and GSDMD levels were dose-dependently upregulated in the TBHP-treated NP cells (Fig. 2i, j). Next, we measured the downstream protein expression levels and found that cleaved CASP-1 and cleaved GSDMD levels were upregulated after TBHP treatment. The ratios of cleaved CASP-1 to pro-CASP-1 and cleaved GSDMD to GSDMD increased dose-dependently after TBHP treatment, quantitatively revealing the increased activation of the NLRP3 inflammasome under oxidative stress (Fig. 2k, l). The enzymatic activity of CASP-1 was determined by measuring the cleavage rate of Ac-YVAD-pNA. We observed a dose-dependent increase in CASP-1 enzymatic activity induced by TBHP (Fig. 2m). In addition, ELISA results indicated that the level of IL-1β in cell culture supernatants were elevated significantly, revealing that oxidative stress promoted inflammatory factor levels in the extracellular microenvironment (Fig. 2n). We used TEM to examine the ultrastructure of NP cells and found cellular swelling with large bubbles in the TBHP-treated NP cells, which is characteristic of pyroptosis induced by the N-terminus of GSDMD^47^ (Fig. 2o). Cell viability was assessed with Hoechst and propidium iodide (PI) staining, which showed significant cell death after TBHP treatment (Fig. 2p). The above data revealed the involvement of NLRP3 inflammasome activation and pyroptosis in the NP cell death under TBHP-induced oxidative stress.

### Inhibiting STING activation alleviated NP cell pyroptosis under oxidative stress

**Fig. 3:**
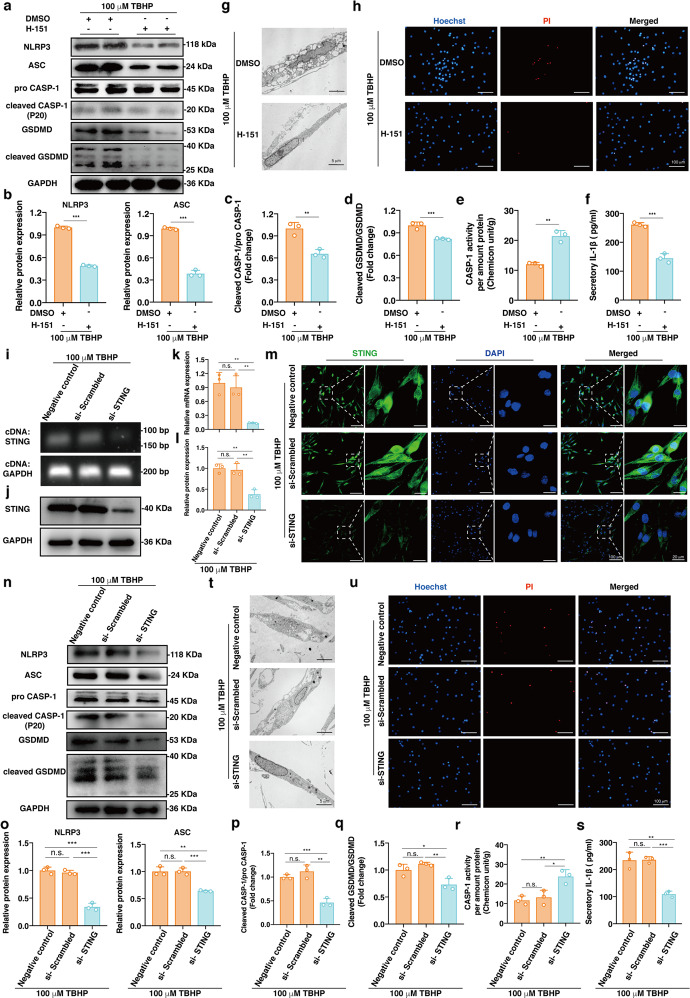
Inhibiting STING activation alleviated NP cell pyroptosis under oxidative stress. A selective small molecule STING antagonist (H-151, 1 μM) or DMSO in combination with 100 μM TBHP was used to treat NP cells for 24 h. **a** Representative western blotting images of NLRP3, ASC, pro-CASP-1, cleaved CASP-1, GSDMD, and cleaved GSDMD in the H-151- or DMSO-treated NP cells. **b** Quantification of the protein levels of NLRP3 and ASC in the H-151-treated NP cells compared to the vehicle control-treated cells. **c** The ratio of cleaved CASP-1 to pro-CASP-1 in the H-151-treated NP cells compared to the vehicle control-treated cells. **d** The ratio of cleaved GSDMD to GSDMD in the H-151-treated NP cells compared to the vehicle control-treated cells. **e** The enzymatic activity of CASP-1 in the H-151- or DMSO-treated NP cells normalized to the total protein amount. **f** The quantitative level of IL-1β in cell culture supernatants in the H-151- or DMSO-treated NP cells. **g** TEM images of the H-151 or DMSO-treated NP cells. **h** Representative fluorescence images of Hoechst/PI staining in the H-151- or DMSO-treated NP cells. NP cells were transfected with STING siRNA and treated with 100 μM TBHP. **i** Representative STING cDNA agar gelatin electrophoresis image in the negative control, si-Scrambled, and si-STING groups. **j** Representative western blotting images of STING after siRNA knockdown. **k** RT-qPCR quantitative analysis of STING mRNA after siRNA knockdown. **l** Quantification of the protein level of STING after siRNA knockdown. **m** Representative immunofluorescence images of STING with different degrees of polymerization after siRNA knockdown. **n** Representative western blotting analysis of NLRP3, ASC, pro-CASP-1, cleaved CASP-1, GSDMD, and cleaved GSDMD after siRNA knockdown. **o** Quantification of the protein expression levels of NLRP3 and ASC after siRNA knockdown. **p** The ratio of cleaved CASP-1 to pro-CASP-1 after siRNA knockdown. **q** The ratio of cleaved GSDMD to GSDMD after siRNA knockdown. **r** The enzymatic activity of CASP-1 after siRNA knockdown normalized to the total protein amount. **s** The quantitative level of IL-1β in cell culture supernatants after siRNA knockdown. **t** TEM images of NP cells after siRNA knockdown. **u** Representative fluorescence images of Hoechst/PI staining after siRNA knockdown. Data are shown as the mean ± SD of at least three independent experiments. Statistical analyses were conducted using two-way ANOVA and Student’s *t* test. The *P* value is indicated by stars: n.s. no significance, *P < 0.05, **P < 0.01, ***P < 0.001.

Previous studies have shown that the cGAS-STING axis increases NLRP3 inflammasome activation in response to pathogenic or damage-associated stress^48,49^. To verify that oxidative stress-induced STING activation was responsible for NLRP3 inflammasome formation and NP cell pyroptotic death, we used a selective human STING antagonist (H-151) to pharmacologically block the activity of STING and treated NP cells with 100 μM TBHP for 24 h^50^. Western blotting results showed that the expression levels of core proteins (NLRP3, ASC, pro-CASP-1, and GSDMD) and effector proteins (cleaved CASP-1 and cleaved GSDMD) of pyroptosis were significantly downregulated in the group treated with the STING antagonist (Fig. 3a, b). In addition, the cleaved CASP-1:pro-CASP-1 ratio, the cleaved GSDMD:GSDMD ratio, and the CASP-1 enzymatic activity were reduced, which indicated reduced activation of the NLRP3 inflammasome (Fig. 3c–e). In addition, the ELISA results indicated that the level of secreted IL-1β in NP cell culture supernatants decreased after STING inhibition (Fig. 3f). TEM showed that inhibition of STING could alleviate TBHP-induced cell swelling and bubble formation (Fig. 3g). The STING antagonist reduced the number of PI/Hoechst double-positive cells and blocked TBHP-induced NP cell death (Fig. 3h). Furthermore, we knocked down STING using small-interfering RNA in NP cells, which were then treated with 100 μM TBHP. We used agar gelatin electrophoresis for visualization and RT-qPCR to quantitatively verify the knockdown efficiency of siRNA against STING at the transcript level (Fig. 3i, k). In addition, western blotting and immunofluorescence showed that the protein expression level of STING was downregulated significantly and that STING knockdown was efficient (Fig. 3j, l, m and Supplementary Fig. 1b). STING knockdown reduced TBHP-induced NLRP3 inflammasome activation and blocked pyroptotic NP cell death (Fig. 3n–u). Collectively, the above data indicated that inhibition of STING could alleviate NLRP3 inflammasome-mediated pyroptosis in NP cells under oxidative stress.

### Oxidative stress-induced mPTP opening and excessive accumulation of cytosolic mtDNA

**Fig. 4:**
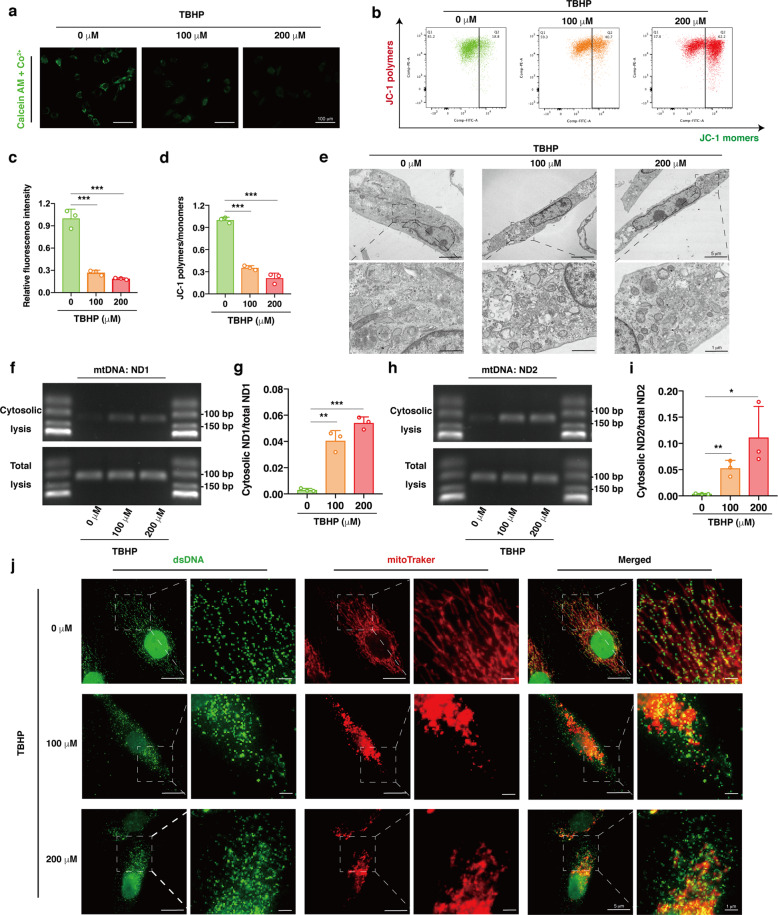
Oxidative stress-induced mPTP opening and excessive accumulation of cytosolic mtDNA. Human NP cells were cultured with 0, 100 or 200 μM TBHP for 24 h. **a**, **c** Representative fluorescence images of calcein AM/Co^2+^ quencher staining and the quantitative analysis of fluorescence intensity for opening levels of mPTP in the NP cells exposed to TBHP compared to the controls. **b**, **d** Representative scatter plots of JC-1 staining and the quantitative analysis of MMP in the TBHP-treated NP cells compared to the controls. **e** Representative TEM images of swelling and vacuolation mitochondria in the TBHP-exposed NP cells. **f**, **h** DNA agar gelatin electrophoresis images of mtDNAs (ND1 and ND2) in cytosolic or total lysates of the TBHP-treated NP cells. **g**, **i** RT-qPCR quantitative analysis of cytosolic mtDNAs (ND1 and ND2) normalized to mtDNAs of total lysates from the TBHP-treated NP cells. **j** Representative fluorescence images of dsDNA (green) and mitochondria (red) in the TBHP-treated NP cells. Data are shown as the mean ± SD of at least three independent experiments. Statistical analyses were conducted using two-way ANOVA and Student’s *t* test. The *P* value is indicated by stars: *P < 0.05, **P < 0.01, ***P < 0.001.

Disturbed mitochondrial homeostasis is a characteristic of IVD degeneration, and mitochondrial damage intrinsically triggers mPTP opening and the subsequent leakage of DAMPs into the cytoplasm, regulating cell death events^33,51,52^. Recent studies have shown increased mPTP opening, cytosolic escape of mtDNA and cGAS-STING axis activation under mitochondrial stress^18,21–24^. To evaluate whether oxidative stress triggers mPTP opening in TBHP-treated NP cells, we performed fluorescence microscopy, confirming a reduction in the amount of calcein-AM retained in the mitochondria, indicating the opening of the mPTP (Fig. 4a, c). In addition, mitochondrial membrane potential (MMP) is maintained by the electrochemical gradient across two mitochondrial membranes, which is reduced by mPTP opening. JC-1 staining revealed a decreased ratio of JC-1 polymers to monomers after TBHP treatment, functionally indicating that oxidative stress enhanced mPTP opening in NP cells (Fig. 4b, d). TEM indicated swelling and vacuolation of mitochondria in the TBHP-exposed NP cells, which is the most prominent characteristic of mPTP opening (Fig. 4e). To further verify the leakage of mtDNA, cytosolic DNA was extracted, visualized by agar gelatin electrophoresis and quantitatively analyzed by RT-qPCR. The results indicated that the levels of ND1 and ND2, which are genes located in mtDNA, were higher in the cytosol of the NP cells exposed to TBHP, while there were no increases in the total levels^18,23^ (Fig. 4f–i). In addition, the immunofluorescence assay indicated increased dsDNA fluorescence intensity outside of mitochondria and nuclei in the NP cells cocultured with TBHP (Fig. 4j). These data suggested that oxidative stress triggered mPTP opening and the excessive accumulation of cytosolic mtDNA, which might contribute to the activation of cGAS-STING-NLRP3 axis-dependent NP cell pyroptosis.

### Pharmacologically blocking mPTP ameliorated activation of the mtDNA-cGAS-STING-NLRP3 axis and NP cell pyroptosis

**Fig. 5:**
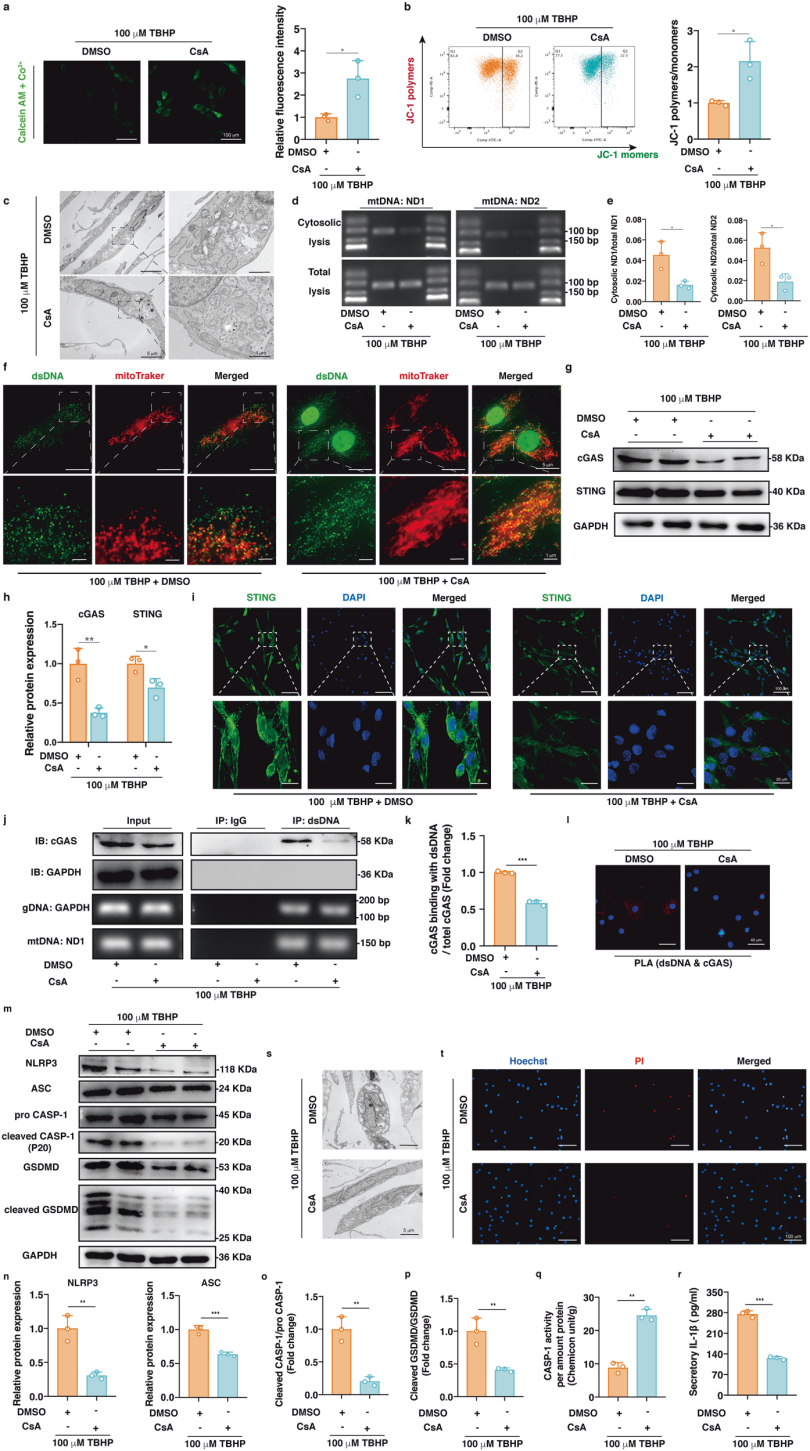
Pharmacologically blocking mPTP ameliorated activation of the mtDNA-cGAS-STING-NLRP3 axis and pyroptosis in human NP cells. Cyclosporine (CsA) or DMSO in combination with 100 μM TBHP was used to treat NP cells for 24 h. **a** Representative fluorescence images of calcein AM/Co^2+^ quencher staining and the quantitative analysis of fluorescence intensity for opening levels of mPTP in NP cells after CsA treatment compared to the controls. **b** Representative scatter plots of JC-1 staining and the quantitative analysis of MMP after CsA treatment compared to the vehicle control. **c** Representative TEM images showing mitochondrial morphology in the CsA- or DMSO-treated NP cells. **d** DNA agar gelatin electrophoresis images of mtDNAs in cytosolic or total lysates from the CsA- or DMSO-treated NP cells. **e** RT-qPCR quantitative analysis of cytosolic mtDNAs normalized to mtDNAs of total lysates from the CsA- or DMSO-treated NP cells. **f** Representative fluorescence images of dsDNA and mitochondria in the CsA- or DMSO-treated NP cells. **g** Representative western blotting images of cGAS and STING in the CsA- or DMSO-treated NP cells. **h** Quantification of the protein expression levels of cGAS and STING in the CsA-treated NP cells compared to the controls. **i** Representative immunofluorescence images of STING with different degrees of polymerization in the CsA- or DMSO-treated NP cells. **j** Representative DNA immunoprecipitation images in the CsA- or DMSO-treated NP cells. **k** Quantification of the enrichment level of cGAS in the DNA immunoprecipitates in the CsA-treated NP cells compared to the controls. **l** The physical interaction between dsDNA and cGAS from in situ PLA images in the CsA- or DMSO-treated NP cells. **m** Representative western blotting images of NLRP3, ASC, pro-CASP-1, cleaved CASP-1, GSDMD, and cleaved GSDMD in the CsA- or DMSO-treated NP cells. **n** Quantification of the protein levels of NLRP3 and ASC in the CsA-treated NP cells compared to the controls. **o** The ratio of cleaved CASP-1 to pro-CASP-1 in the CsA-treated NP cells compared to the controls. **p** The ratio of cleaved GSDMD to GSDMD in the CsA-treated NP cells compared to the controls. **q** The enzymatic activity of CASP-1 in the CsA- or TBHP-treated NP cells. **r** The quantitative level of IL-1β in cell culture supernatants after CsA or DMSO treatment. **s** TEM images of the CsA or DMSO-treated NP cells. **t** Representative fluorescence images of Hoechst/PI staining in the CsA- or DMSO-treated NP cells. Data are shown as the mean ± SD of at least three independent experiments. Statistical analyses were conducted using two-way ANOVA and Student’s *t* test. The *P* value is indicated by stars: *P < 0.05, **P < 0.01, ***P < 0.001.

To further confirm that mPTP opening and mtDNA escape into the cytosol were essential for cGAS-STING-NLRP3 axis activation and NP cell pyroptosis, we treated NP cells with cyclosporine (CsA), a pharmacological inhibitor of mPTP opening, which significantly abrogated TBHP-induced mPTP opening and decreased MMP, morphological mitochondrial damage and cytosolic mtDNA accumulation (Fig. 5a–f). In addition, CsA decreased the protein expression levels of cGAS and STING, the formation of STING polymers, the enrichment of cGAS in DNA immunoprecipitates, and PLA signals between dsDNA and cGAS (Fig. 5g–l and Supplementary Fig. 1c). Moreover, TBHP-induced NLRP3 inflammasome activation and NP cell pyroptosis were inhibited by CsA (Fig. 5m–t). These data suggested that oxidative stress-induced mPTP opening and mtDNA leakage were responsible for cGAS-STING-NLRP3 axis activation and NP cell pyroptosis.

### Suppressing mPTP opening and STING activation ameliorated IVD degeneration in vivo

**Fig. 6:**
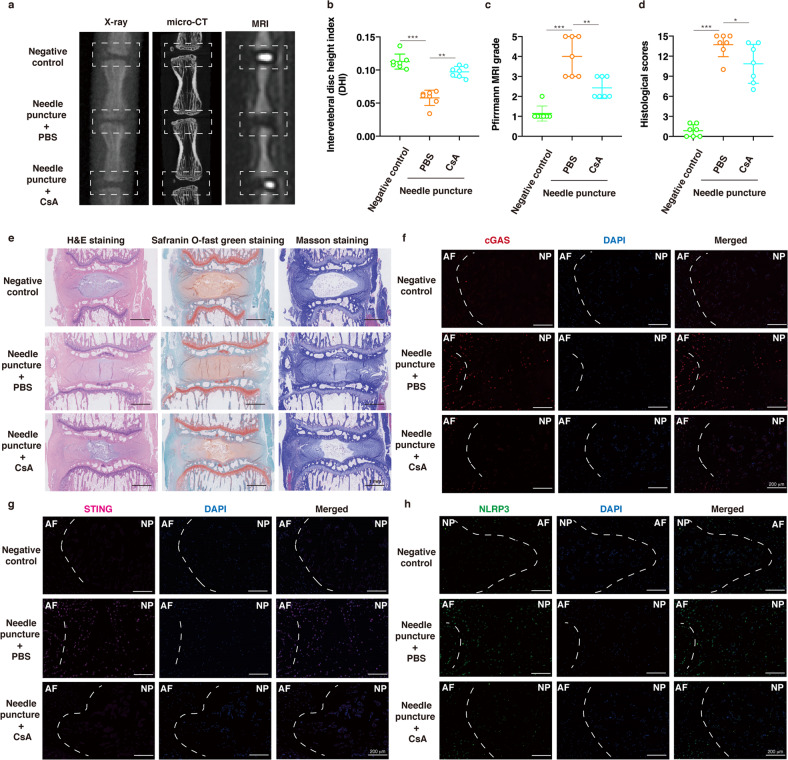
Suppressing mPTP opening ameliorated IVD degeneration in vivo. A needle puncture-induced IVD degeneration model on Co8/9 and Co9/10 discs was established, and the punctured Co9/10 discs were immediately intradiscally injected with CsA (100 μM) every week for one month, while other punctured Co8/9 discs were injected with the same amount of PBS every week for one month. **a** Representative X-ray, micro-CT, and T2-weighted MRI images of a rat tail. **b** The disc height index (DHI) was measured in the negative control, needle puncture + PBS injection, and needle puncture + CsA injection groups (*n* = 7). **c** The Pfirrmann MRI grade scores were evaluated in the three groups (*n* = 7). **d** The histological grades were assessed in the three groups (*n* = 7). **e** Representative H&E staining, safranin O-fast green staining, and Masson staining of rat discs in different groups. **f**–**h** Representative fluorescence images of cGAS, STING, and NLRP3 in the three groups. Data are shown as the mean ± SD of at least three independent experiments. Statistical analyses were conducted using two-way ANOVA and Student’s *t* test. The *P* value is indicated by stars: *P < 0.05, **P < 0.01, ***P < 0.001.

**Fig. 7:**
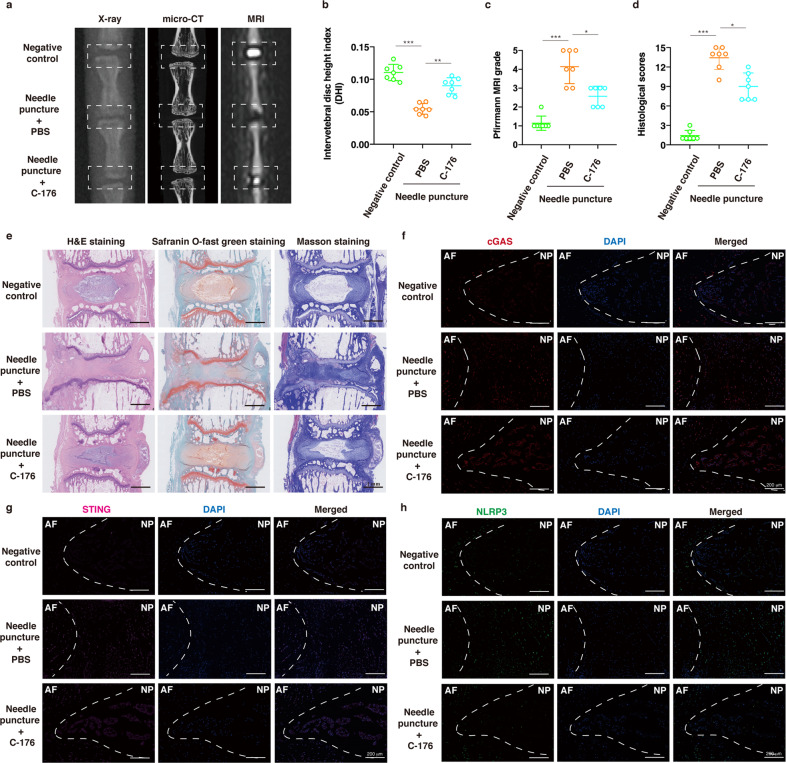
Suppressing STING activation ameliorated IVD degeneration in vivo. A needle puncture-induced IVD degeneration model on Co8/9 and Co9/10 discs was established, and the punctured Co9/10 discs were immediately intradiscally injected with C-176 (an inhibitor of STING, 10 μM) every week for one month, while other punctured Co8/9 discs were injected with the same amount of PBS every week for one month. **a** Representative X-ray, micro-CT, and T2-weighted MRI images of a rat tail. **b** The disc height index (DHI) was measured in the negative control, needle puncture + PBS injection, and needle puncture + C-176 injection groups (*n* = 7). **c** The Pfirrmann MRI grade scores were evaluated in the three groups (*n* = 7). **d** The histological grades were assessed in the three groups (*n* = 7). **e** Representative H&E staining, safranin O-fast green staining and Masson staining of rat discs in different groups. **f**–**h** Representative fluorescence images of cGAS, STING, and NLRP3 in the three groups. Data are shown as the mean ± SD of at least three independent experiments. Statistical analyses were conducted using two-way ANOVA and Student’s *t* test. The *P* value is indicated by stars: *P < 0.05, **P < 0.01, ***P < 0.001.

To further evaluate the therapeutic efficacy of pharmacological inhibition of mPTP opening and STING activation in vivo, we established a disc percutaneous needle puncture animal model of IVD degeneration using Sprague-Dawley rats. After one month, X-ray and micro-CT analyses confirmed that the disc heights in the needle puncture group that was treated with PBS collapsed more than those in the group that was treated with CsA or C-176 (a selective rat STING antagonist) (Figs. 6a, b, 7a, b). MRI examination showed that the signal intensities of the IVD after PBS injection were not homogeneous and that the T2-weighted signals were lower than those in the group that received CsA or STING antagonist injection (Figs. 6a, c, 7a, c). H&E staining, safranin O-fast green staining and Masson staining showed a loose well-organized NP tissue structure with star-shaped cells, increased tissue fibrillation in the NP area, and no distinguishable boundary between NP and AF tissue. Conversely, CsA or STING antagonist administration reduced IVD histological degeneration compared with PBS injection (Figs. 6e, 7e). In addition, the histological scores in the IVD degeneration with needle puncture group were significantly increased compared with those of the CsA or STING antagonist injection group (Figs. 6d, 7d). Immunofluorescence staining showed that CsA reduced the fluorescence intensities of cGAS, STING and NLRP3, and the STING antagonist decreased the fluorescence intensity of NLRP3 but not cGAS or STING compared to PBS injection (Figs. 6f–h, 7f–h). These data demonstrated that pharmacological inhibition of mPTP opening and STING activation could ameliorate damage in the needle puncture rat model of IVD degeneration.

## Discussion

**Fig. 8:**
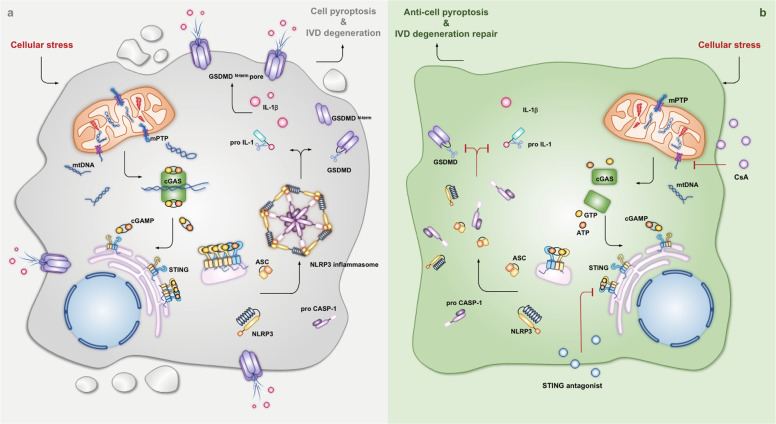
Cytosolic escape of mitochondrial DNA triggers cGAS-STING-NLRP3 axis-dependent nucleus pulposus cell pyroptosis. Graphical abstract of the crucial roles of cytosolic escape of mtDNA via mPTP opening and cGAS-STING-NLRP3 axis-dependent nucleus pulposus cell pyroptosis in intervertebral disc degenerative progression. **a** Cytosolic escape of mtDNA via mPTP opening triggers cGAS-STING-NLRP3 axis-dependent nucleus pulposus cell pyroptosis during intervertebral disc degenerative progression. **b** Antagonists of mPTP opening (CsA) and STING (C-176 and H-151) alleviate nucleus pulposus cell pyroptosis and intervertebral disc degeneration via inhibition of the cGAS-STING-NLRP3 axis.

IVD degeneration is a common musculoskeletal disorder frequently associated with LBP and causes a heavy socioeconomic burden^1,2^. Although numerous etiological factors are involved in IVD degenerative progression, including heredity, aging, and biomechanical overloading, the degenerative IVD progression has not been fully elucidated^8,53^. Unfortunately, the current diagnostic techniques and therapeutic methods do not reduce LBP-related disability^7,54^. Importantly, our data showed that the expression of cGAS, STING and NLRP3 was associated with the degree of degeneration of NP tissues. Oxidative stress promoted activation of the cGAS-STING axis and NLRP3-mediated NP cell pyroptosis. In addition, STING inhibition and knockdown significantly alleviated oxidative stress-induced NP cell pyroptosis by inhibiting the NLRP3 inflammasome. Mechanistically, we found that oxidative stress disturbed mitochondrial homeostasis, which triggered mPTP opening and excessive cytosolic mtDNA accumulation. In addition, pharmacologically inhibiting mPTP opening by CsA administration alleviated activation of the cytosolic mtDNA-cGAS-STING-NLRP3 axis and NP cell pyroptosis in vitro and the progression of degeneration in vivo (Fig. 8). Collectively, we demonstrated that the cytosolic mtDNA-cGAS-STING-NLRP3 axis might be a potential indicator to predict the degree of IVD degeneration.

Regardless of etiological factors, IVD degeneration is associated with common disease phenotypes: the progressive loss of NP cells, the secretion of proinflammatory mediators, inflammatory microenvironment formation, and declines in cellular functions and biomechanical properties^8,53^. The key to understanding IVD degeneration is how NP cells sense pathogenic or damage-associated signals and respond to these signals, leading to cell dysfunction and microenvironmental imbalance. The cGAS-STING axis senses dislocated DNA to trigger an innate immune response via the IFN cascade or the NF-κB pathway in many degenerative diseases^17–19^. mPTP opening is a consequence of diverse cellular stresses that provide a gateway for mtDNA to overcome two barriers (the inner and outer mitochondrial membranes) and translocate into the cytosol^20^. Previous studies have shown that disturbances in mitochondrial homeostasis lead to proapoptotic protein release during degenerative IVD progression, while few studies have focused on mtDNA escape^33,51,52^. Tian et al.^34^ reported that cGAS and STING were associated with the degenerative progression of IVD. In this study, we verified that cytosolic mtDNA acts via the mPTP under oxidative stress and acts as a DAMP to trigger the STING-NLRP3 axis and promote NP cell pyroptosis. Although the physiological functions of the mPTP have been well established, the molecular components of the mPTP remain unkown^55^. Recently, Santhanam et al. showed that the mPTP is a heterooligomeric protein complex consisting of VDAC, SPG7, and CypD, and blocking SPG7-CypD binding alleviated oxidative stress-induced cell death^56^. Numerous studies have verified that pharmacological inactivation of the mPTP prevents mtDNA leakage and cytosolic mtDNA accumulation through inhibition of CypD or VDAC oligomerization. Recent studies involving the mPTP have focused on VDAC and CypD dysfunction, but it is not clear how mitochondrial stress affects the interaction of mPTP components and whether other proteins participate in mPTP opening to release mtDNA into the cytosol^18,19^.

The NLRP3 inflammasome is a heterooligomeric protein complex that is involved in surveillance of pathogenic motifs, damage signals, and microenvironmental perturbations^25^. Recent studies have shown that aberrant NLRP3 activation under pathogenic or damage-associated stress could drive inflammation in the IVD to modulate degenerative progression^32,46^. Due to the similarities in the cGAS-STING signaling axis and NLRP3 inflammasome in response to cellular stress as well as the downstream effects, recent studies have focused on their relationship. IFN cascades induced by cGAS increase CASP-1 and CASP-11 activation as well as pyroptotic cell death during *Chlamydia trachomatis* infection^57^. Human myeloid cells under DNA stimuli activate the cGAS-STING axis rather than the AIM2 inflammasome, as STING activation and translocation to lysosomes trigger lysosomal cell death with activation of the NLRP3 inflammasome^49^. In addition, the cytosolic DNA-cGAS-STING axis promotes lipopolysaccharide-induced acute lung injury by modulating the NLRP3 inflammasome in macrophages^48^. In this study, we investigated whether mtDNA rather than exogenous DNA, as an endogenous damage signal under oxidative stress, was sensed by cGAS and regulated NLRP3 inflammasome-mediated pyroptotic NP cell death, which was more consistent with the pathology of IVD degeneration. During cell apoptosis, oxidized mtDNA is generated and released from mitochondria to activate the NLRP3 inflammasome^39^. However, our data do not exclude the possibility of a direct interaction between cytosolic oxidized mtDNA and the NLRP3 inflammasome in NP cells, as direct binding might similarly be inhibited by reducing mPTP opening and oxidized mtDNA leakage. Notably, many forms of DNA, including long dsDNA, single-stranded DNA with local secondary structure, synthetic DNA, and oxidized DNA, activate cGAS, while NLRP3 is preferentially activated by oxidized DNA rather than normal DNA, according to current research results^16,39^. The AIM2 inflammasome is another kind of inflammasome that senses cytosolic DNA damage and leads to pyroptotic cell death^58^. Our data do not deny the possible role of AIM2 under oxidative stress. Some studies have shown that the cGAS-STING axis can detect lower levels of cytosolic DNA than AIM2^59^. Therefore, the factors that determine which DNA sensor is activated depending on the levels of cytosolic DNA and which factors affect the strength of signaling activated by cytosolic DNA or the duration of signaling remain unclear^60^. Importantly, we described the crucial role of the cGAS-STING axis in self-DNA sensing and NLRP3 inflammasome-dependent cell pyroptosis.

In summary, our findings provide MRI and histological evidences to show that cGAS, STING, and NLRP3 are associated with the progression of IVD degeneration. This study also highlights the crucial involvement of the cGAS-STING signaling axis in NLRP3 inflammasome-mediated NP cell pyroptosis under oxidative stress. Mechanistically, we found that oxidative stress-induced mPTP opening and mtDNA leakage into the cytosol, which triggered the activation of cGAS-STING. Furthermore, pharmacologically inhibiting mPTP opening and STING activation alleviated IVD degenerative progression in vivo. Thus, the cGAS-STING-NLRP3 axis could not only be a potential indicator to predict the degree of degeneration but also be a promising target to improve the disease-modifying treatments of IVD degeneration and LBP.

## Supplementary information

Supplementary materials

## Data Availability

The datasets generated during the current study are not publicly available due to the data also forming part of our ongoing study but are available from the corresponding author on reasonable request.
