# Supplementary material for: Cytosolic escape of mitochondrial DNA triggers cGAS-STING-NLRP3 axis-dependent nucleus pulposus cell pyroptosis
Source: Exp Mol Med. 2022 Feb 10;54(2):129–42. doi: 10.1038/s12276-022-00729-9 (PMC8894389; doi:10.1038/s12276-022-00729-9)
Supplement: Supplementary file 1 — Supplementary materials [file 12276_2022_729_MOESM1_ESM.pdf]

Supplementary Table 1. Information of participants

Case Number	Gender	Age (Years)	Diagnosis	Segment	MRI Grade	Operation Method
Case 1	Male	32	Lumbar disc herniation	L4-L5	IV	Percutaneous endoscopic transforaminal discectomy
Case 2	Male	15	Idiopathic scoliosis	L1-L2	I	Open spine surgery
Case 3	Male	31	Lumbar disc herniation	L5-S1	III	Percutaneous endoscopic transforaminal discectomy
Case 4	Female	18	Lumbar disc herniation	L5-S1	II	Percutaneous endoscopic transforaminal discectomy
Case 5	Male	45	Lumbar disc herniation	L5-S1	III	Percutaneous endoscopic transforaminal discectomy
Case 6	Female	46	Lumbar disc herniation	L5-S1	IV	Percutaneous endoscopic transforaminal discectomy
Case 7	Male	22	Lumbar disc herniation	L3-L4	II	Percutaneous endoscopic transforaminal discectomy
Case 8	Male	58	Lumbar disc herniation	L5-S1	IV	Percutaneous endoscopic transforaminal discectomy
Case 9	Female	14	Idiopathic scoliosis	T12-L1	I	Open spine surgery
Case 10	Male	28	Lumbar disc herniation	L4-L5	II	Percutaneous endoscopic transforaminal discectomy
Case 11	Male	20	Idiopathic scoliosis	L1-L2	I	Open spine surgery
Case 12	Female	52	Lumbar disc herniation	L5-S1	IV	Percutaneous endoscopic transforaminal discectomy
Case 13	Female	42	Lumbar disc herniation	L4-L5	III	Percutaneous endoscopic transforaminal discectomy
Case 14	Female	12	Idiopathic scoliosis	T11-T12	I	Open spine surgery
Case 15	Male	27	Lumbar disc herniation	L5-S1	II	Percutaneous endoscopic transforaminal discectomy
Case 16	Female	44	Lumbar disc herniation	L5-S1	III	Percutaneous endoscopic transforaminal discectomy

Supplementary Table 2. Information for antibodies

Antibodies	Source	Identifier
anti-cGAS	Proteintech	26416-1-AP
anti-STING	Proteintech	19851-1-AP
anti-NLRP3	Proteintech	19771-1-AP
anti-ASC	Proteintech	10500-1-AP
anti-CASP-1 / anti-cleaved CASP-1	Proteintech	22915-1-AP
anti-GSDMD / anti-cleaved GSDMD	Proteintech	20770-1-AP
anti-GAPDH	Proteintech	60004-1-AP
anti-rabbit IgG, HRP-linked antibody	Cell Signaling Technology	7074
anti-mouse IgG, HRP-linked antibody	Cell Signaling Technology	7076
anti-dsDNA	Abcam	Ab27156
IgG isotype control monoclonal antibody	Proteintech	66360-2-Ig

Supplementary Table 3. Table of materials for western blotting

Reagent	Source
Polyvinylidene difluoride membrane (PVDM)	Bio-Rad
5% Bovine serum albumin (5% BSA)	Boster
0.1% Tris-buffer saline containing 1:1000 Tween-80 (0.1% TBST)	Boster

Supplementary Table 4. Primers used in this study

Gene Name	Forward Primer	Reverse Primer
Homo-STING	5’-CACTTGGATGCTTGCCCTC-3’	5’-GCCACGTTGAAATTCCCTTTTT-3’
Homo-GAPDH	5’-CAAGAAGGTGAAGCAGG-3’	5’-TCAAAGGTGGAGGAGTGGGT-3’
si-STING	5’-GCAUCAAGGAUCGGGUUUA-3’	5’-UAAACCCGAUCCUUGAUGCTT-3’
si-Scrambled	5’-UUCUCCGAACGUCACGUTT-3’	5’-ACGUGACACGUUCGGAGAATT-3’
Homo-ND1	5’-CTCTTCGTCTGATCCGTCCT-3’	5’-TGAGGTTGAGGTCTGTTAGT-3’
Homo-ND2	5’-GTAGACAGTCCCACCCTCAC-3’	5’-TTGATCCCGTTTCGTGCAAG-3’

Supplementary Table 5. The parameters of radiography images

**X-ray**			
Collimator-to-film distance	66 cm	Exposure	63 mAs
Penetration power	35 kV		
**Micro-CT**			
Voltage	70 kV	Current	200 µA
Resolution	18 µm	Exposue time	300 ms
**MRI (Fast-spin echo sequence)**			
Time-to-repetition	2000 ms	Time-to-echo	36 ms
Matrix	256 (h) × 256 (v)	Field of view	6.00/3.00 cm
Flip angle	180°		

Supplementary Table 6. Information of assays/instruments/software used in this study

Methods	Critical commercial assays/Instruments/Software	Source
Tyramide signal amplification immunofluorescence	Opal 4-Color Manual IHC Kit	PerkinElmer
RNA reverse transcription	HiScript III 1st Strand cDNA Synthesis Kit	Vazyme
General PCR	2×Phanta Master Mix	Vazyme
RT-PCR	ChamQ SYBP Color aPCR Master Mix (High ROX Premixed)	Vazyme
Cytosolic mtDNA purification	TIANamp Genomic DNA Kit	TIANGEN
Flow cytometer	FACSCablibur flow cytometer	BD Biosciences
Microscope	Fluorascence microscope	Olympus, BX53, Melville, USA
Proximity ligation assy	Duolink® In situ PLA® Kit	SigmaAldrich
Transmission electron microscope	Techai G2 TWIN TEM	FEI
Three-dimensional reconstructions of micro-CT	NRecon software version 1.5	Skyscan, Belgium

Supplementary Fig. 1 The representative immunofluorescence images and quantitative analysis of fluorescence intensity of STING

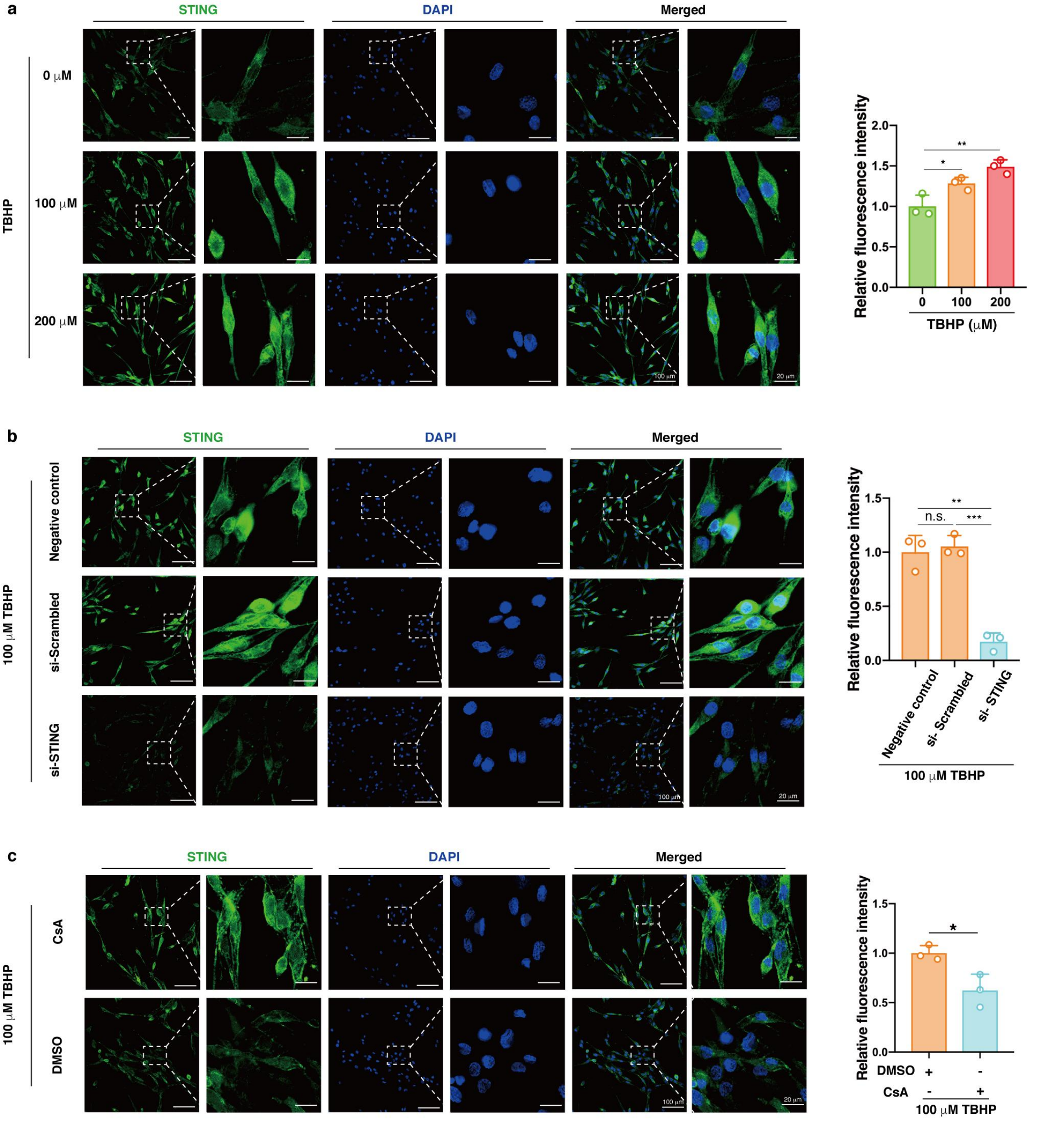

a Representative immunofluorescence images of STING with different degrees of polymerization and the quantitative analysis of fluorescence intensity of STING in the TBHP-treated NP cells.

b Representative immunofluorescence images of STING with different degrees of polymerization and the quantitative analysis of fluorescence intensity of STING after siRNA knockdown.

c Representative immunofluorescence images of STING with different degrees of polymerization and the quantitative analysis of fluorescence intensity of STING in CsA or DMSO-treated NP cells after exposed 100 μm TBHP.

Statistical analyses were conducted using two-way ANOVA and student's t-test. *P* value was descried by stars: n.s. no significance, * $P < 0.05$, ** $P < 0.01$, *** $P < 0.001$.
